# GRANDPARENTS RAISING GRANDCHILDREN IN KOREA: CULTURAL UNDERSTANDING OF MULTIGENERATIONAL CAREGIVING

**DOI:** 10.1093/geroni/igz038.1804

**Published:** 2019-11-08

**Authors:** Youjung Lee, Sok An

**Affiliations:** 1 Binghamton University, Binghamton, New York, United States; 2 Korea Rural Economic Institute (KREI), Naju-si, Korea, Republic of

## Abstract

Korean grandparents raising their grandchildren play significant roles as social and family resources in Korea. They experience caregiving stress that negatively influences their mental health while struggling with limited social support from peers, and minimal respite care and community resources. Despite a need for resources and information concerning this growing population, there is limited knowledge on Korean custodial grandparents, especially the distinctive cultural factors influencing their multigenerational caregiving experiences. This qualitative study explored Korean custodial grandparents’ experiences of raising grandchildren and cultural meanings of multigenerational caregiving. Using a phenomenological approach, semi-structured interviews with 23 custodial grandparents were conducted from December 2018- January 2019 in two urban and three rural places in Korea. Among the grandparents, five were grandfathers. The mean age of the grandparents was 72 years old (range: 61-82), and the mean age of their grandchildren was 13 years old (range: 1-21). On average, the grandparents had raised 1.5 grandchildren (range: 1-3) for 11 years (range: 1.5-20). Four key themes emerged from the general structure of the lived experiences of Korean grandparents: a) intensive child care support from the grandchildren’s uncle and aunt; (b) stigma of a child’s divorce and custodial grandparenting; (c) different social expectations toward maternal grandparents; and (d) increased needs on financial educational support for grandchildren. The finding reinforces the significant function of collectivistic and family-based Asian culture in understanding experiences of Korean custodial grandparents. It also highlights society’s view of only men carrying forward family lineage and its implications on grandparents’ perceptions about multigenerational caregiving.

